# Production and Surface Modification of Cellulose Bioproducts

**DOI:** 10.3390/polym13193433

**Published:** 2021-10-07

**Authors:** Sumedha Liyanage, Sanjit Acharya, Prakash Parajuli, Julia L. Shamshina, Noureddine Abidi

**Affiliations:** Fiber and Biopolymer Research Institute, Texas Tech University, Lubbock, TX 79409-5019, USA; sumedha.liyanage@ttu.edu (S.L.); sanjit.acharya@ttu.edu (S.A.); prakash.parajuli@ttu.edu (P.P.); jshamshi@ttu.edu (J.L.S.)

**Keywords:** cellulose, surface functionalization, biomaterials, bioproducts, applications

## Abstract

Petroleum-based synthetic plastics play an important role in our life. As the detrimental health and environmental effects of synthetic plastics continue to increase, the renewable, degradable and recyclable properties of cellulose make subsequent products the “preferred environmentally friendly” alternatives, with a small carbon footprint. Despite the fact that the bioplastic industry is growing rapidly with many innovative discoveries, cellulose-based bioproducts in their natural state face challenges in replacing synthetic plastics. These challenges include scalability issues, high cost of production, and most importantly, limited functionality of cellulosic materials. However, in order for cellulosic materials to be able to compete with synthetic plastics, they must possess properties adequate for the end use and meet performance expectations. In this regard, surface modification of pre-made cellulosic materials preserves the chemical profile of cellulose, its mechanical properties, and biodegradability, while diversifying its possible applications. The review covers numerous techniques for surface functionalization of materials prepared from cellulose such as plasma treatment, surface grafting (including RDRP methods), and chemical vapor and atomic layer deposition techniques. The review also highlights purposeful development of new cellulosic architectures and their utilization, with a specific focus on cellulosic hydrogels, aerogels, beads, membranes, and nanomaterials. The judicious choice of material architecture combined with a specific surface functionalization method will allow us to take full advantage of the polymer’s biocompatibility and biodegradability and improve existing and target novel applications of cellulose, such as proteins and antibodies immobilization, enantiomers separation, and composites preparation.

## 1. Introduction

As the ubiquity of synthetic plastic waste and its harmful effects on the environment, aquatic and terrestrial life, and human health increase, the concept of making materials from renewable natural sources has become popular. The magnitude of plastic pollution is vast, and it has become one of the most pressing societal concerns. Only around 9% of the 6300 metric tons of plastics made in 2015 have been recycled, 12% incinerated, and the rest has accumulated in the environment [1]. Much of this problem emerges from excessive consumption of single-use plastics as they are convenient, affordable, and easily accessible. Plastic waste accumulation also increases due to the lack of economically attractive and energy-efficient recycling options and almost indefinite shelf-life of most plastics. If the current production and recycling trends persist, scientists predict that approximately 12,000 metric tons of plastics will accumulate globally by 2050 [1]. A world without plastics seems unimaginable, given their convenience and diversity in applications. Therefore, in order to protect nature without compromising the needs of the present and future generations, environmental scientists are searching for novel bio-based plastics that are recyclable, compostable, or biodegradable as well as for cost-effective and energy-efficient plastic waste processing technologies.

Numerous studies have demonstrated that using green technologies for the production of versatile materials can reduce carbon footprint. Use of natural polymers as synthetic plastic substitutes could represent a viable solution for most aforementioned problems. Among natural polymers, cellulose extracted mainly from cellulosic and lignocellulosic biomass, such as cotton, wood, and crop residues are of substantial importance and has the potential to fulfill the growing demand for bio-based plastics. Cellulose is a renewable natural polymer of significant commercial importance that represents ~1.5 × 10^12^ tons/year of total biomass [2]. It is the most abundant natural polymer on Earth. Cellulose is biosynthesized by plants, species of bacteria (*genera Acetobacter*, *Agrobacterium*, and *Sarcina ventriculi*), and algae (*Rhodophyta phylum* and *Phaeophyceae* class) [3,4]. The cellulose content in cotton is as high as 90%, and in wood is 40–50% [5,6].

**Figure 1:**
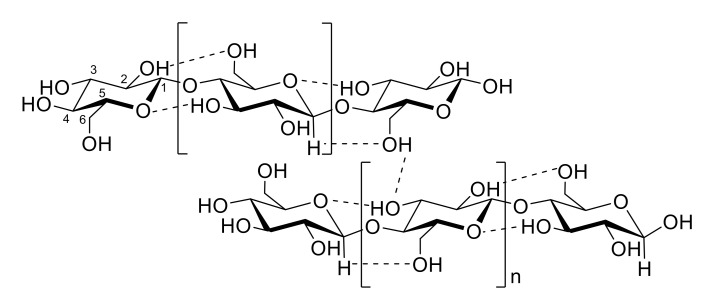
Intra-chain and inter-chain hydrogen bonding in cellulose.

Cellulose has a structural formula (C_6_H_10_O_5_)_n_, where n ranges from 10,000 to 15,000 [7], and is composed of D-glucose units covalently linked together by 1 → 4 glycosidic bonds (i.e., beta linkages) [8]. Because of a large number of hydroxyl groups present in the polymer, it is extensively hydrogen-bonded, having both inter- and intra-chain bonding. Intra-chain hydrogen bonding, which is responsible for stabilization of the glycosidic bonds, is formed between O(3)-H…O(ring) and O(2)-H…O(6). Inter-chain hydrogen bonding, which is responsible for parallel stacking of multiple cellulose chains occurs between O(3)-H…O(6) (Figure 1) [9]. 

Cellulose fiber has a defined hierarchical order in its organization. Native cellulose fiber consists of several microfibrils (5–10 nm in diameter each, depending on plant tissue and species) bundled together. In turn, microfibrils are made of stacked semi-crystalline elementary fibrils. Sequentially, elementary fibrils, with dimensions of 3.5 × 5.3 nm each, are assembled from glucan chains (generally, 36) [10]. Bacterial cellulose (BC) is obtained by a build-up of nanofibers. 

Cellulose is a highly crystalline polymer [2,11,12], and naturally exists in allomorphs I_α_ (triclinic unit cell) and I_β_ (monoclinic unit cell), with the I_α_/I_β_ ratio dependent on cellulose origin [13]. In addition, other allomorphs of cellulose are known: cellulose II, III, and IV. The cellulose II allomorph can be formed from cellulose I by alkali treatment (mercerization) or through dissolution–regeneration technique. Typically, cellulose has a high molecular weight and its degree of polymerization (DP) depends on a cellulose source and method of polymer isolation. For cotton, DP ranges from ~800–10,000 [14,15,16]. One of the important properties of cellulose is its biodegradability, a breakdown by microorganisms into carbon dioxide and water, such that it can be consumed by the environment; it has been shown that cellulose disintegrates completely in compost at an ambient temperature within 4 months [2,17]. 

However, despite many valuable properties and environmental benefits of cellulose, its transformation into usable forms with desired characteristics and functionalities requires several steps, namely purification, dissolution, preparation of required material architectures, and dehydration. Unlike synthetic plastics, cellulose does not melt when subjected to heat, and therefore, cellulose dissolution is of foremost importance among all the steps [17]. During this process, the crystalline structure of natural cellulose is disrupted, and the polymer can be transformed into different forms during the regeneration process. At the same time, an extensive hydrogen bonding network between neighboring cellulose molecules and highly crystalline microfibril structures that give unique physicochemical characteristics to plants make it recalcitrant to dissolution in water and in most common organic solvents. 

**Figure 2:**
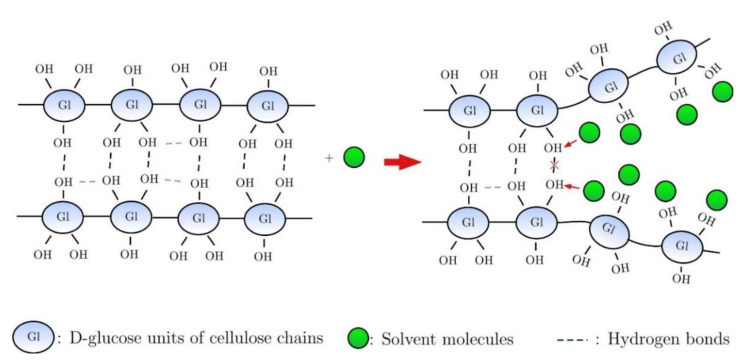
Schematic of cellulose dissolution.

There is a long and fruitful history of cellulose dissolution, and highly effective solvent systems for dissolution of cellulose have already been reported, which include *N*-methylmorpholine-*N*-oxide (NMMO), aqueous NaOH/urea, *N*,*N*-dimethylacetamide/LiCl (DMAc/LiCl), and ionic liquids (ILs, the salts that remain liquids at or below 100 °C [18]); these systems are comprehensively discussed elsewhere [15]. Among these, ILs efficiently dissolve cellulose at relatively low temperatures and are considered to be green solvents for cellulose. During the last two decades, numerous ILs with different structural characteristics have been synthesized, and their dissolution mechanism and the influence of constituent ions, and cosolvents on cellulose dissolution capacity have been extensively studied [19,20,21,22,23,24]. The dissolution mechanism typically involves breaking hydrogen bonds between the neighboring cellulose molecules [17] (Figure 2), resulting in a molecularly dispersed solution [25] or a colloidal dispersion of several hundred cellulose chains [12], capable of forming new hydrogen bonds during regeneration. Thus far, it has been challenging to find an ideal solvent that does not react with cellulose nor break cellulose chains [25]. 

It is well known that the degree of polymerization (DP), crystallinity (CrI), and crystallite size of cellulose depend on its origin (e.g., wood cellulose vs. cotton cellulose). While these parameters impart unique fascinating characteristics to cellulose, they hamper its dissolution. Therefore, frequently, conditions are optimized for effective dissolution of cellulose obtained from various sources. For example, cotton cellulose that possesses a high DP of 8000–15,000 and CrI of ~80% is recalcitrant to dissolution under mild conditions, although these characteristics are cultivar-dependent [26,27]. The dissolution process is affected by several factors such as cellulose drying conditions (oven drying vs. freeze drying), various pretreatments, cellulose concentration, dissolution temperature, pH, and moisture content [12,16,28]. Upon dissolution, viscous cellulose solutions can be converted into different material architectures using dry- or wet- jet spinning [29,30,31], electrospinning [32,33,34], or casting, followed by gelation, regeneration in protic solvents, such as water and alcohols, and drying (*via* air-drying, hot pressing, supercritical CO_2_ drying, etc.) [2,17,24,25,26,27,28]. Each of these techniques results in cellulose recovery in a form of amorphous bioproducts (e.g., hydrogels, films/membranes, aerogels, and beads [35,36,37,38,39]). These bioproducts manifest many unique characteristics of natural cellulose and their functionality is limited to the presence of hydroxyl groups.

Although cellulose is an economical and available resource that offers a wide range of physical and chemical properties, often its properties are not suitable for a specific application. With the growth of the bioplastic industry, it became apparent that modification of polymeric products is essential for producing genuine bioplastics that can compete with highly variable synthetic polymers. Thus, instead of modifying the polymer’s bulk properties, surface modifications of cellulose and cellulose composites (e.g., surface coating, chemical modification, plasma or vapor deposition treatment, a combination of such), tailored for a specific purpose, can result in materials with targeted properties. For instance, cellulose is hydrophilic, and impartment of hydrophobicity is required in applications such as self-cleaning [40] or self-healing materials [41], oil and water separation [42], electromagnetic interference (EMI) shielding [43], etc. Recent review discusses superhydrophobic modification of cellulose and potential applications [44]. Likewise, although nanocelluloses are often employed as reinforcing fillers owing to their impressive mechanical strength, tuning of their interfacial properties through incorporation of various functional groups is required to improve its dispersibility in common solvents [45]. The ability to improve the properties of cellulose through surface modification points to a promising future for cellulosic biomaterials in many fields. 

In the current review, we revisited the most recent developments in surface modification of cellulose-based bioproducts. The review also highlights targeted development of novel cellulosic products and their applications, with a specific focus on certain material architectures: hydrogels, aerogels, beads, membranes, and nanomaterials. The selection of material architecture combined with specific surface functionalization will allow us to take full advantage of the polymer’s biocompatibility and biodegradability and improve existing and target novel applications of cellulose. Because the topic is broad, we have focused on the type of bioproducts, product characteristics, and surface functionalization techniques for specific use.

## 2. Surface Modification

The surface of a material represents the boundary between the bulk polymer and the outer environment, and the structural and chemical changes that occur on the surface tailor them for different applications. A wide range of surface modification techniques, such as physicochemical, biological, and mechanical methods, have been developed for modifying polymer surfaces. Some of the most common surface modification techniques that have been used for tailoring cellulosic surfaces are summarized herein. 

### 2.1. Plasma

Plasma, sometimes referred to as the fourth state of matter [46], is an ionized gas and can be defined as a medium consisting of gaseous or fluid-like mixtures of a wide range of charged particles, such as free electrons, ions, radicals, and metastable molecules. In a typical process, plasma is obtained by exposing a gas to radiofrequency, microwave, or electrons from a hot filament discharge [47,48,49]. Mostly, plasmas are divided into two groups, namely equilibrium or thermal/hot plasma and non-equilibrium or nonthermal/cold plasma. Extremely high temperatures (e.g., 20,000 K for He) are required to generate hot plasma, and all ionized species are in thermodynamic equilibrium. In cold plasma, the gas remains at room temperature. Since extremely high temperatures of the thermal plasma is detrimental to polymeric materials, the use of plasma technique for polymer surface modification is limited to cold plasmas [47,48]. 

In practice, plasma is generated by applying an electric field to electrodes with a gas in between them, either at atmospheric or reduced pressure. The properties of plasma depend on the nature of gases, electric power, and material and geometry of electrodes. Typical gases used for surface modification are He, Ar, O_2_, N_2_, NH_3_, and CH_4_. The interaction between the surface of a material and plasma leads to either direct surface modification through introduction of new functional groups or indirect modification. For example, the use of reactive NH_3_ plasma leads to the introduction of amine groups while O_2_ plasma introduces a mixture of oxygen containing functional groups (e.g., COOH and OH). In indirect modification methods, plasma (He or Ar) introduces free radicals on the surface, which are typically used for cross-linking or surface grafting of other molecules with desired functionalities [48,50]. A thin layer of a surface coating can also be applied by depositing a thin polymer film on the substrate, which is polymerized from organic, organo-silicone, or organometallic monomers introduced into the plasma system. This type of plasma surface modification strategy is called plasma polymerization. The polymerization can be initiated either in vapor phase or at the surface [48,50]. 

Plasma technique is a clean, dry, and energy efficient technique that offers advantages over other surface modification methods (i.e., wet chemical methods) [51]. This is especially true given the commercialization of industrial plasma equipment. In addition, use of plasma treatment has lower global warming potential values (GWP), acidification potential values (AP), and other life cycle analysis (LCA) parameters than those associated with “traditional” methods. Moreover, the effect of plasma is limited to less than 100 nm from the surface [51], and no significant degradation and subsequent mechanical degradation of the substrate occur [50,52]. Another important benefit of plasma technique is that, irrespective of the geometry, the surface is uniformly modified. Furthermore, this technique can be applied to complex objects such as artificial organs, nanoparticles, films, and highly porous materials such as cellulose aerogels [53,54,55,56]. The major challenges for large-scale industrial plasma treatment involve 3D aspect and often large surface area of the materials that need to be treated, as well as the necessity of a continuous process.

The interaction of the active plasma species with cellulosic textiles (e.g., cotton, linen) can either remove particles from the surface (e.g., surface cleaning, etching, sterilization) or add particles to the substrate (e.g., activation, functionalization, or coating), and is possible at different stages of the textile production—at sliver, yarn, or fabric level. Surface activation typically refers to the formation of oxygen-containing groups on the surface, such as –OH, =O,–COOH, and is not permanent. Functionalization refers to grafting of chemical groups onto the surface, and plasma coating refers to the nanometer-thick deposition of a coating onto the substrates. The most common treatments of textile include modification of the surface hydrophobicity/hydrophilicity, adhesive properties, enhancing textile printability and dyeability, imparting of antibacterial properties, fire retardant, or anti-shrink agents onto the textile surface, etc. [57]

### 2.2. Surface Grafting

A graft copolymer typically consists of one or more branches of one polymer attached to the backbone of another polymer. In general, graft copolymerization is of particular interest because of its versatility in creating a wide range of functional groups on the surface since the process allows for combining desirable properties of two or more polymers into a single physical unit. Furthermore, the properties of a copolymer can be tailored by altering different parameters (e.g., polymer type, degree of polymerization, and polydispersity of the main and side chains as well as the graft density and the graft uniformity) [58,59]. Different categories of surface grafting include plasma-induced grafting, radiation-induced grafting, photochemical grafting, chemical grafting, and enzymatic grafting [60,61]. Among different types of chemical grafting, reversible-deactivation radical polymerization (RDRP, also known as living/controlled radical polymerizations) [62], ionic grafting, and ring-opening polymerization (ROP) [61] are employed the most often. 

RDRP methods allow synthesizing functional polymeric hybrid biomaterials with precisely designed polymeric structure; these materials exhibit properties significantly different from those of the base polymer. Such grafting of synthetic polymers onto the cellulose backbone typically is able to achieve a high level of graft density. Based on the reaction mechanism, RDRPs are classified into reversible deactivation methods and reversible transfer methods (reversible addition-fragmentation chain transfer (RAFT)). Reversible deactivation methods include nitroxide-mediated radical polymerization (NMP) and atom transfer radical polymerization (ATRP). 

There are three types of grafting approaches described in the literature for the synthesis of graft copolymers of cellulose. In the most commonly used “grafting from” approach, the initiator is grafted onto the carbohydrate backbone and is used to polymerize from the site on the backbone. On the contrary, “grafting to” approach is characterized by coupling the reactive end group of a preformed polymer with functional groups present on the cellulose backbone, i.e., a pre-made polymer is linked to a complementary functionality on the backbone. The third, “grafting through”, approach involves the copolymerization of usually a vinyl macromonomer of cellulose with the co-monomer [58,59]. The “grafting from” approach offers an important advantage in terms of allowing easy access of the reactive groups to the chain ends of growing polymers, thereby leading to high graft density. On the contrary, limited graft density is the inherent outcome of the “grafting to” approach, because of steric hindrances encountered by to-be-grafted long polymer chains at the surface [58]. 

Graft copolymerization of many monomers onto a cellulose backbone has been effectively employed for its surface chemical modification. Few extensive reviews on using RDRP methods for the synthesis of hybrid materials based on cellulose have been published, and both “grafting from” and “grafting to” approaches have been extensively reviewed [63,64]. Thus, synthetic polymers, usually those made of vinyl and acrylic monomers, have been grafted onto cellulose substrates of different types [59], such as fabrics, nanofibrous surfaces, and cellulose nanocrystals, imparting important functional properties: hydrophobicity [50,65], stimuli responsiveness [66], etc. For instance, Abidi et al. successfully imparted hydrophobic properties to cotton fabrics by graft copolymerization of cellulose and vinyl monomers [67].

Modifications of wood-based materials by ATRP methods were recently examined by Zaborniak et al., who synthesized graft co(polymers) starting with pure cellulose, cellulose derivatives, and cellulosic materials (e.g., membranes) [68]. Cellulose esterified with *α*-bromoisobutyryl bromide (BriBBr), Cell-Br, was used as cellulose-based ATRP initiator. Most of the reactions required the use of copper salts/amine-type ligands catalysts. The grafted synthetic polymers included acrylic acid (AA), methyl methacrylate (MMA), 2-(dimethylamino)ethylmethacrylate (DMAEMA), sodium 4-styrenesulfonate (NASS), N-isopropylacrylamide (NIPAM), glycidyl methacrylate (GMA), etc. Examples of so-called “simplified electrochemically mediated ATRP” (seATRP) were also reported for preparation of brush-shaped block copolymer with a dual hydrophilic poly(acrylic acid)-block-poly(oligo(ethylene glycol) acrylate) (PAA-b-POEGA) arms [69].

Supplemental activator and reducing agent atom transfer radical polymerization (SARA ATRP) method is one more method to be used for a sophisticated design of graft copolymers. Copolymer based on rigid and hydrophilic cellulose and flexible and hydrophobic polyisoprene, combining rigidity and flexibility, hydrophobicity and hydrophilicity, all in a single macromolecule, was synthesized from cellulose derivative [70].

NMP-mediated radiation graft copolymerization using nitrogen oxide compounds to trap reactive polymer radicals produced by ionizing radiation, and subsequent living polymerization with vinyl monomers, is typically conducted on cellulose acetate or halogenated cellulose. Thus, cellulose acetate-g-polystyrene (CA-g-PS) grafted copolymers have been synthesized by NMP under homogeneous conditions using the 1,2-intermolecular radical addition with the BlocBuilder MA (N-[1-diethylphosphono-(2,2-dimethylpropyl)]nitroxide based alkoxyamine) initiators [71].

Finally, RAFT-mediated “grafting from” approach from pure cellulose surfaces was reported for, e.g., the synthesis of cellulose-g-pHEMA copolymers under γ-irradiation. Such “grafting from” ATRP strategy allows the preparation of high molecular weight graft copolymers consisting of a cellulose main chain with acrylate copolymer side chains. Authors achieved control of molecular weight and distribution of grafted chains and graft ratios up to 92% (*w*/*w*) [72]. Several other polymerization methods, such as ROP to copolymerize ringed monomers (e.g., ε-caprolactone and lactide), have also been used [73,74].

### 2.3. Chemical Vapor Deposition

Chemical vapor deposition (CVD) is a process where precursors in the gaseous phase react to form a thin solid film of specified properties on the surface of a substrate. Both gas phase and surface reactions can be involved in the process of transferring precursors to the film [75]. CVD has been well-established as a powerful technique for creating inorganic surfaces, and it is the predominant technology for growing high-quality inorganic layers [76,77]. CVD is widely employed in the production of high-purity bulk and powder materials and the deposition of materials on a surface [78]. This technique can be used to obtain well-adhered conformal coatings with a thickness less than 100 nm for different technological applications [76]. Since recently, with the development of solvent-free CVD polymerization techniques such as oxidative CVD (oCVD) and initiated CVD (iCVD), it became possible to modify a diverse array of substrates (e.g., organic or inorganic, rigid or flexible, planar or three-dimensional, and dense or porous) with functional polymers. Since such vapor deposition techniques are usually performed at low substrate temperatures and without solvents, CVD techniques are particularly beneficial for fragile substrates that do not resist to heat or solvents such as plastic sheets, papers, and vulnerable electronic structures [76,79,80]. Several studies have employed CVD techniques for surface modification of various types of cellulose substrates to impart hydrophobic properties, i.e., bamboo fabric [80], bacterial cellulose membrane [81], and nanocellulose aerogels [82]. Furthermore, researchers have successfully used CVD to obtain electrically conductive coatings on cellulose substrates [83].

### 2.4. Atomic Layer Deposition

Atomic layer deposition (ALD) is a sophisticated technique of depositing ultra-thin films of a few nanometers in a precisely controlled fashion. The ALD technique is similar to CVD and is considered to be a special variant of CVD because gaseous precursors are injected to the reaction chamber which then form a desired material due to chemical surface reactions. However, unlike CVD, general ALD processes are characterized by sequential alternating pulses of highly reactive gaseous precursors, one at a time, that react with the substrate [84]. A typical ALD cycle consists of four distinct steps: (1) pulse of the first gaseous precursor and its chemisorption onto the substrate, (2) purging of excess of the precursor along with the reaction byproducts using inert gas, (3) pulse of the second gaseous precursor and its reaction with the adsorbate formed by the first precursor, and (4) purging of the excess of the second precursor along with formed reaction byproducts using inert gas. This cycle is repeated several times until the desired thickness of the deposited layer is achieved. Advantageous features such as conformality, large area uniformity, precise thickness control, and reproducibility make ALD a superior technique. However, the slowness of the process is a major drawback [84,85]. The ALD technique is widely used in many important industrial applications (e.g., protective coatings, microelectronics, photovoltaics, and solid oxide fuel cells) [86]. Studies have shown that ALD can be successfully employed to impart functional coatings of metal oxides and metal nitrides onto different types of cellulose based substrates such as cotton fibers [87,88], viscose fibers [88], and nanocellulose films [89].

Along with these techniques, researchers have successfully employed many other surface modification procedures, such as physical vapor deposition (e.g., sputter coating), printing, immersion, padding, exhaustion, and well-known sol–gel process to alter cellulosic surfaces [90,91]. Importantly, the sol–gel process is considered as a feasible and versatile approach to modify textile surfaces with a variety of active compounds. 

## 3. Surface Modification of Cellulose and Cellulose Bioproducts 

### 3.1. Surface Modification of Textile Materials

Surface modification of cellulose is also important in textile materials. Since surface modification improves wearer comfort and fabric performance, it is of profound importance to the textile industry. The textile industry reports the most traditional cellulose functionalization for clothing, carpeting, automotive, agricultural, and medical applications. Importantly, surface functionalization improves softness, dyeability, and wettability of textiles made from natural and regenerated cellulose fibers and introduces additional functionalities, such as easy-care finishing, antimicrobial activity, flame retardancy, self-cleaning capability, UV protection, and water repellency [91]. 

For instance, antimicrobial textile finishes have been increasingly used as they prevent the growth and the spread of pathogenic microbes and offer protection to the wearer and textile. The widely used antimicrobial agents for textiles include chitosan, metal salts, halogenated phenols, and N-halimanes, which can be applied to the fabric surface using conventional pad-dry-cure method, spraying, exhaustion, foam-application, and surface grafting. In particular, metals and metal oxide nanoparticles (e.g., silver, zinc oxide, and titanium dioxide) are extensively used for surface functionalization of cotton fabrics to impart antimicrobial activity, UV protection [92,93,94,95,96], and photocatalytic self-cleaning behavior [97,98,99,100]. Superhydrophobicity (lotus effect) is also imparted to the fabric surface for improved self-cleaning behavior, and liquid droplets that fall onto modified surfaces tend to entrap soil/dirt particles and roll off the fabric surface [91,98]. Water, oil, and liquid repellency have been successfully imparted to cotton fabrics by surface coating/grafting with hydrophobic compounds (e.g., vinyl laurate, oleic acid, and fluorinated compounds) via pad-knife-pad coating [101], nanoparticle and molecular vapor deposition (NVD and MVD, respectively) [98,102,103], plasma-induced grafting [50,104], and plasma-enhanced chemical vapor deposition [105]. Airoudj et al. reported that surface functionalization with a fluorinated polymer layer introduced Janus wetting behavior, where one side of the fabric had superhydrophobic properties while maintaining hydrophilic properties on the opposite side [105]. Qin et al. produced superhydrophobic flame retardant cotton fabrics by surface coating with diammonium phosphate solution, followed by surface adhesion of modified ZrO_2_ [106]. Nie et al. prepared environmentally benign flame retardant and hydrophobic cotton fabrics by surface modification with non-toxic bio-based phytic acid and n-Dodecyltrimethoxysilane [107]. 

**Figure 3:**
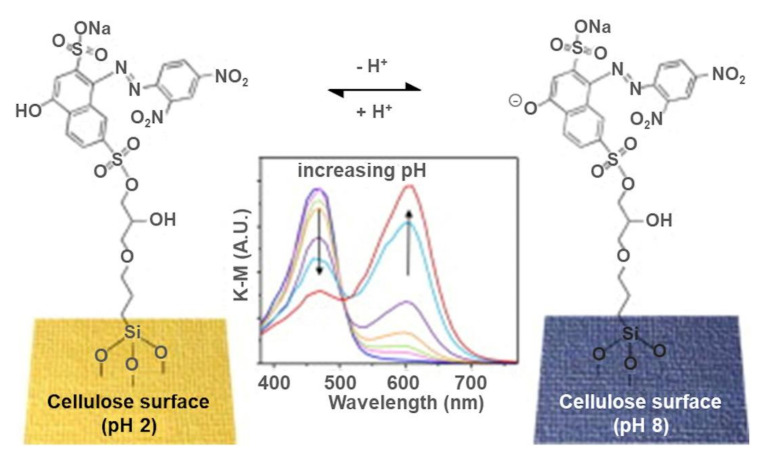
pH sensitive cotton fabrics prepared by covalent immobilization of Nitrazine Yellow (NY) containing hybrid matrix on the textile surface. Reprinted from ref. [111] © 2021 with permission from Elsevier.

Since cellulose is widely used in medical textiles, surface functionalization is crucial in preventing cross-infections, disease transmissions, and growth and spreading of pathogenic microbes. Song et al. produced superhydrophobic and photocatalytic cotton fabrics that suppressed blood adhesion (liquid repellency) and improved bacterial repellency and bactericidal effects, suggesting potential use in biomedical fields [108]. Montaser et al. functionalized cotton gauze fabrics through cationization and incorporation of antimicrobial agents and drug molecules, followed by coating with salicyl-amine-chitosan biopolymer for improved wound healing efficacy [109]. Additionally, there is a rapid development of stimuli-responsive or smart textile materials to monitor real-time and continuous system changes in biomedical (e.g., wound healing), defense, aerospace, and protective clothing. Smart textiles show reversible color change triggered by external stimuli, including temperature, pH, and light. For instance, pH sensitive cotton fabrics have been developed by photo-grafting of modified halochromic dye (i.e., Nitrazine yellow (NY) dye with a methacrylate group) [110], immobilizing NY dye using the sol–gel method [111], and sol–gel impregnating halochromic resorufin and 3-glycidoxypropyltrimethoxysilane hybrid sol [112] (Figure 3). 

### 3.2. Surface Modification of Cellulose Bioproducts

Cellulose based bioproducts are manufactured through dissolution of the polymers in specific solvents followed by transformation into desired products (e.g., via casting, 3D printing, extrusion), regeneration, and drying. Both solvent systems and coagulants have a strong influence on mechanical and surface characteristics of the regenerated materials [25]. Despite the countless studies that have been conducted to date, cellulose dissolution and regeneration are fundamental aspects of producing cellulose-based materials, and therefore, still remain active research fields. Additionally, the potential uses of cellulose bioproducts depend critically on their surface characteristics, suggesting that emphasis should be placed on modifying their surface chemistry. A wide range of different approaches can be applied to impart additional functionalities to cellulose surfaces using previously discussed techniques. Emphasis herein will be placed on the production of cellulose bioproducts and their direct surface modification targeting specific applications. 

#### 3.2.1. Porous Cellulose Products 

Cellulosic materials that hold air within their porous network represent an important class of cellulose bioproducts. Often called cellulose aerogels, they not only possess the fascinating properties of cellulose, but also integrate other important properties of porous materials, such as high porosity, high surface area, and low density. The abundance of hydroxyl groups over a large surface area allows introducing several functional groups onto cellulose chains which renders these porous bioproducts useful for targeted applications (e.g., provides more active sites for heterogeneous catalytic reactions) [113]. Cellulose aerogels include natural cellulose aerogels, regenerated cellulose aerogels, and aerogels of cellulose derivatives of both cellulose I and II crystalline structures [114]. Natural cellulose aerogels are produced by direct drying of cellulose dispersions, whereas regenerated cellulose aerogels are developed through dissolution of the polymer in a proper solvent system, regeneration in anti-solvent, and drying. Cellulose derivative aerogels are obtained through similar preparation methods, but because cellulose derivatives are soluble in volatile organic solvents (VOCs), regeneration in anti-solvent/solvent-exchange steps are often omitted [114]. 

Cellulose hydrogels are highly porous materials that can absorb, retain, and release water in a reversible manner [115]. Cryogels are prepared by solvent exchange followed by freeze drying of cellulose gels and known to possess high surface area (190–213 m^2^/g) and porosity characteristics [116]. Drying technique remains a principal factor determining the porosity and pore characteristics of porous cellulose products, however casting process, nature of solvents and antisolvents, and inclusion of additives, such as surfactants, inorganic salts, metal oxides, etc. can affect their surface characteristics. 

#### 3.2.2. Cellulose-Based Hydrogels

Cellulose hydrogels are usually prepared through casting cellulose solutions into a mold followed by gelation and regeneration in water or another anti-solvent. Hydrogels range from physical gels (made by polymer chain self-entanglement) to chemically cross-linked networks. The main common attribute of hydrogels is a gel-like network containing both a liquid phase (usually water) and a solid phase. For hydrogels preparation, typically, cellulose solution is cast into a suitable mold, and subsequently gelated under different temperatures. Hydrogel thickness is controlled by the mass of the film-forming solution poured into a mold [117]. Thus, Chang et al. successfully prepared cellulose-based hydrogels using molding, by dissolving carboxymethylcellulose (CMC)/cellulose in NaOH/urea aqueous system containing epichlorohydrin (ECH) cross-linker, followed by keeping the solution at different temperatures (i.e., heating at 60 °C for 12 h, 50 °C for 20 h or freezing at −20 °C for 20 h) [118,119]. Another technique is casting, in which a solution is placed onto a glass plate and spread with a roller or casting knife on a glass substrate and subsequently gelated at ambient temperature [120] or directly immersed into a coagulating bath [116,121]. Two important categories of regenerated cellulose gels, namely hydrogels and alcogels, can be prepared by regenerating the gel in an aqueous or an alcohol medium [121]. Hydrogels and alcogels are dried as needed using an appropriate drying technique, such as reduced pressure/vacuum drying [120,122] and freeze drying [116,121]. 

**Figure 4:**
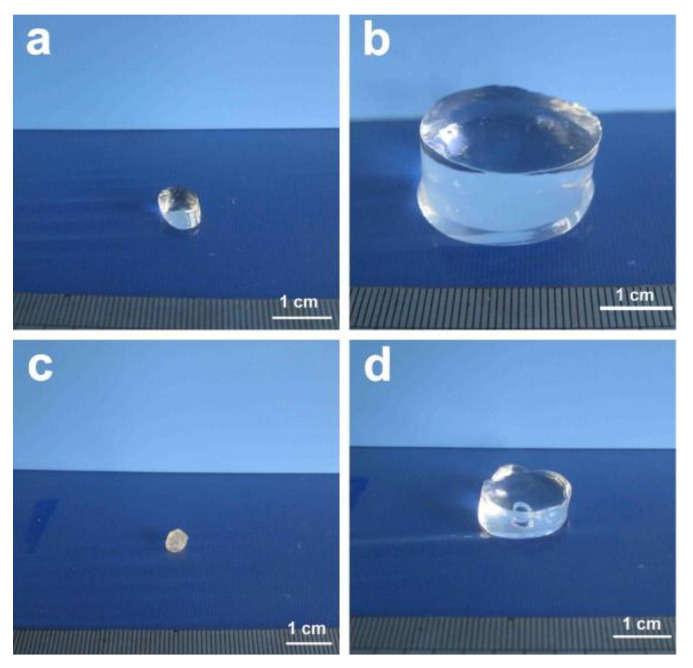
Photographs of superabsorbent cellulose/carboxymethylcellulose (CMC) hydrogels: (**a**) original hydrogel, (**b**) swollen hydrogel, (**c**) dried hydrogel, and (**d**) hydrogel after swelling in NaCl solution for a week. Reprinted from ref. [118] © 2021 with permission from Elsevier.

It has been reported that the surface area and capillarity, crucial for most of the targeted applications, depend on the hydrogel microstructure [123]. For example, cellulose-based hydrogels loaded with bovine serum albumin (BSA) exhibited a swelling/reswelling ratio of 1000, controlled release of embedded BSA, and smart swelling and shrinking in NaCl or CaCl_2_ aqueous solution [118] (Figure 4). Owing to their hydrophilicity, biocompatibility, stimuli-sensitivity, and exceptional adsorption capability, cellulose-based hydrogels have found promising applications in a diverse range of fields, including biomedical, pharmaceutical, water purification, and wastewater treatment [115]. 

There have been many reports on the surface modification of cellulose-based hydrogels for different applications. Thus, Isobe et al. used several aqueous and alcoholic coagulants to prepare gels that were found to have different internal surface polarity, which in turn affected the absorbent behavior of the hydrogels [121]. The authors reported that hydrogels coagulated in alcohols showed better affinity for a Congo red azo-dye than hydrogels that were coagulated in water. 

Kim and Kuga grafted polyallylamine onto commercial-grade cellulose gels to increase the ion-exchange capacity targeting chromatographic applications [124]. To introduce additional surface functionalities onto cellulose hydrogels, several researchers have used functionalized cellulose polymer as the starting material. For example, RodrÍguez et al. produced cationic cellulose hydrogels by crosslinking two cationic hydroxyethycelluloses [125]. The resulting cationic hydrogels showed significant drug loading capacity and pH-/ion-sensitive release of the anionic amphiphilic drug, diclofenac sodium. Way et al. showed that surface modified cellulose nanocrystals (CNCs) could be employed to introduce additional functionalities to the corresponding suspensions, gels, and composites, imparting properties such as sensitivity to external stimuli (e.g., pH sensitivity) [126]. Ali et al. reported that the irradiation of carboxymethyl cellulose (CMC)-based hydrogels during crosslinking and gelation steps increased their swelling behavior and induced pH-dependent drug release, possibly due to the chain scission of the polymer network [127]. 

#### 3.2.3. Aerocellulose Monoliths

**Figure 5:**
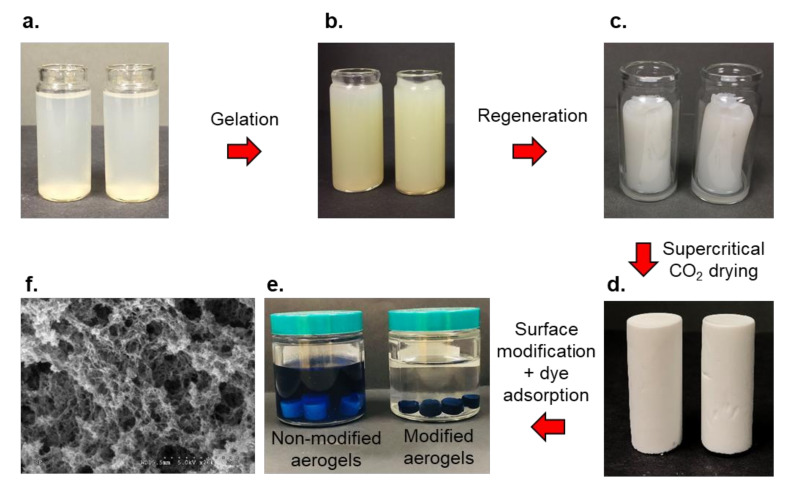
Preparation of cellulose aerogel monoliths. (**a**) cellulose solution in glass molds, (**b**) gelated cellulose solution, (**c**) regenerated cellulose hydrogel, (**d**) supercritical dried cellulose aerogel monoliths (aerocellulose monoliths), (**e**) surface modified and non-modified cellulose aerogels in dye solutions (the non-modified samples have not adsorbed dye from the solution and the aerogels have retained their original color. The surface modified samples have adsorbed dye from the solution, and the solution has become clear, and aerogels have become dark blue due to dye adsorption), and (**f**) SEM micrographs of an aerocellulose monolith cross-section.

Monoliths are single-piece solid cellulosic materials with highly interconnected pores, high surface area, low density, and low thermal conductivity [128]. In addition to unique characteristics of porous materials discussed above, monoliths are mechanically robust, have controllable pore size, and do not have a void volume [129]. Moreover, the convection mode of mass transport adds a significant advantage to the monoliths. Monoliths exhibit higher hydrodynamic porosity than the packed particle beds, implying that the pore structures of monoliths are readily available for the fluid flow than the packed particles where the flow is restricted to interstitial spaces [130]. They are commonly prepared using the sol–gel process, regeneration, and drying [38] (Figure 5). In this process, cellulose solution is poured into a glass mold, gelated at 50 °C for 2 h, regenerated in water, solvent-exchanged with acetone, and subsequently dried using supercritical drying technique [37,38,39], during which the overall volume and network structure of the gel remain largely unchanged. Owing to high porosity and high specific surface area (100 to 400 m^2^/g [38,39,131,132]), ultra-lightweight aerocellulose monoliths with tunable morphological properties (e.g., pore characteristics, shape, size), and inherent beneficial properties of cellulose, are attractive candidates for various applications. Specifically, cellulose monoliths can be shaped into different forms, including microfluidic channels, micropipette tips, and capillary columns [133].

Cellulose monoliths allow easy functionalization for important applications, including chromatographic and separation technologies [129]. For example, Xin et al. introduced different functional groups on the cellulose backbone, targeting molecular immobilization and separation [134]. The epoxy group was introduced via reaction with epichlorohydrin in NaOH solution followed by conversion into amine- and aldehydes-functionalized aerogels by reacting with 1,2-bis(2-aminoethoxy)ethane (BAE) and glutaraldehyde, respectively. Similarly, Pan et al. introduced anion exchange and pseudo affinity (sulfated cellulose) functionalities onto cellulose monoliths to separate influenza virus (H1N1) [135]. The authors reported that the monolith with pseudo-affinity demonstrated better virus separation efficiency and less DNA and protein retention compared to that of non-modified monoliths. In another study, CVD technique was used to coat the aerogel with fluorinated silane (1H,1H,2H,2H-perfluorodecyltrichlorosilane, PFOTS) [128]. The authors reported that the modified aerogel had high oleophilic properties as indicated by the contact angles of castor oil and hexadecane. Similar to other porous cellulose materials, functionalized aerocellulose monoliths offered a promising approach to wastewater treatment and water purification [39,136]. Hu et al. functionalized cellulose monoliths with 3-chloro-2hydroxypropyl) trimethylammonium chloride to introduce positively charged sites for removal of dye molecules from wastewater [39]. The authors reported that the presence of numerous quaternary ammonium cations in the functionalized samples made them highly efficient in immobilizing negatively charged dye compared to that of control samples.

#### 3.2.4. Cellulose Beads

**Figure 6:**
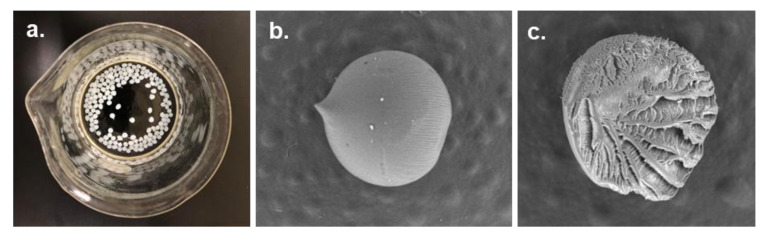
Cellulose beads (CBs) prepared by dropping technique. (**a**) CBs in a coagulation bath, (**b**) SEM micrograph of a CB, and (**c**) SEM micrograph of a CB cross-section.

Cellulose beads (CBs) are smaller-sized particles prepared through the usual route of dissolution of cellulose or cellulose derivatives followed by shaping into a spherical form, sol–gel transition, drying, and fine-tuning of pore characteristics [137]. Particularly, shaping is the most crucial step in CBs preparation, which is usually achieved either by dispersion techniques or dropping the cellulose solution into a coagulating bath using droplet forming machines such as atomizers and jet cutting machines [138]. In the dropping technique, beads are formed by the solidification of cellulose solution by adding it drop-wise into an aqueous coagulating medium (Figure 6). During this process, the combined force of gravity and pressure contribute to the formation of small droplets of cellulose solution pressurized through a narrow orifice or perforated material [139]. The shape and size of cellulose beads could be affected by several factors, including falling height, diameter of the orifice, ejection speed, solution concentration, and coagulation bath [140]. In the dispersion technique, the cellulose solution is dispersed into an immiscible solvent of opposite polarity [141]. Generally, the beads obtained by the dispersion technique are smaller than those obtained by dropping and are in the range of 10–100 µm. However, the mixing speed, viscosity of the cellulose solution, dispersion medium, and the ratio of hydrophobic to hydrophilic solvent affect the size of beads [137].

CBs can be either porous or nonporous. Porous CBs are characterized by high specific surface area and low density. Often named as microspheres, pellets, and beaded cellulose or pearl cellulose, they range from several microns to few millimeters in size [137]. Since CBs reduce the backpressure under flow conditions, they are more advantageous than films or other irregularly shaped particles [142]. 

There has been extensive research work carried out on the surface functionalization of CBs for various types of applications. Thus, CBs and surface modified CBs have found numerous applications in chromatography, drug loading and control release, protein immobilization, and wastewater treatment [137,143]. Xiong et al. imparted hydrophobicity through the reaction of cellulose with trimethylsilyl chloride or hexamethyldisilazane for size exclusion chromatography (SEC) applications [144]. Similarly, Peksa et al. performed carboxymethylation of cellulose beads with chloroacetic acid for ion-exchange applications [145]. Zhang et al. modified porous cellulose beads with amino functional groups to adsorb and separate hyperin and 2’-O-galloylhyperin from the pyrola extract [146]. The authors reported that the modified cellulose beads had three times higher adsorption capacities compared to that of commercial resins. Ruan et al. prepared chitosan crosslinked 2,3-dialdehyde cellulose beads for chemical waste treatments [142]. The authors obtained a fast and efficient Congo red dye adsorption and desorption at pH 2 and 12, respectively, indicating a great potential for chemical waste treatment. 

#### 3.2.5. Films and Membranes

Cellulose films and membranes are sheet-like thin and flexible materials regenerated from cellulose solutions through casting, followed by gelation, regeneration, and dehydration. Usually prepared in either porous or non-porous forms, cellulose films and membranes represent an important class of cellulose bioproducts. Importantly, the ease of preparation, the excellent mechanical properties, and the easy functionalization make them high-value cellulosic materials with widespread applications. Owing to excellent chemical stability, high tenacity in the wet state, biocompatibility, and biodegradability, cellulose films and membranes have found a wide range of applications ranging from simple packaging material, ultrafiltration devices, smart materials to membrane separation (e.g., ultrafiltration, dialysis, and fractionation of polymer mixtures) [147]. 

**Figure 7:**
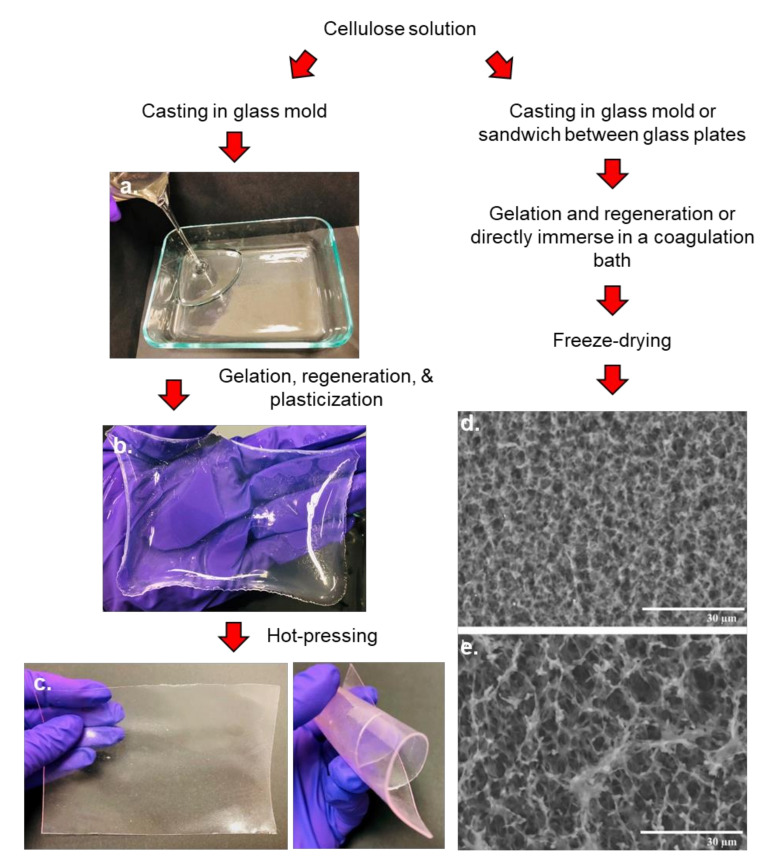
Production of non-porous and porous cellulose films via casting approach. (**a**) Casting cellulose solution in a glass mold, (**b**) regenerated and plasticized cellulose hydrogel, (**c**) hot-pressed flexible cellulose films, and porous cellulose films (**d**) free surface and (**e**) fracture surface (Figure 7d,e were reprinted from Ref. [16] with permission from wileyonlinelibrary.com).

In general, films and membranes are developed by casting a thin layer of cellulose solution onto a suitable substrate [16,28,35] and subsequently gelating it in ambient temperature conditions. In addition, a thin layer of cellulose solution can be deposited on glass substrates by slowly dipping glass slides in the solution [148] or by spin-coating [149]. Instead of gelation, cellulose layer deposited on a glass substrate can also be air dried [148], vacuum dried [149], or directly immersed into a protic solvent [150,151,152,153,154,155,156] and regenerated in water to make hydrogels, which are later dried using an appropriate technique (i.e., air, vacuum or freeze drying). Many parameters, including solvents and antisolvent, membrane thickness, additives such as surfactants, and temperature of the coagulation bath affect the properties of the membranes [152,153,155,157]. Cellulose films with homogenous porous structures and mesh networks have been also developed by coagulating cellulose solutions in different coagulating baths (e.g., H_2_SO_4_, H_2_SO_4_/Na_2_SO_4_, Na_2_SO_4_, HOAc, and (NH_4_)_2_SO_4_) [152,158]. Recent studies also reported that heat-pressing of hydrogels between two metal plates results in transparent, uniform, and strong films [36,159]. While hot pressing generates non-porous and smooth films, the freeze-drying process preserves the membrane pore structures (Figure 7). Plasticization (with e.g., glycerol) improves elongation and flexibility of cellulose-based films [17,159]. 

Several studies reported on the preparation of porous cellulose membranes for different applications. For example, Khare et al. prepared porous cellulose films by casting the cellulose solution onto a glass plate and regenerating in acetone followed by solvent exchange [151]. Acharya et al. prepared porous cellulose films by gelating, regenerating, and freeze-drying a cellulose solution sandwiched between glass slides [16]. 

Similar to other cellulose-based products, the manipulation of the physical and chemical properties is crucial for films and membranes in tailoring their properties for various applications. Fernandes et al. introduced antimicrobial properties by grafting aminoalkyl groups on porous cellulose films [160]. The authors reported that the antimicrobial cellulose membranes prepared by grafting aminoalkyl groups using 3-aminopropyltrimethoxysilane (APS) had excellent antimicrobial activity against *S. aureus* and *E. coli* and good biocompatibility, which made them suitable for tissue engineering, wound healing, and as tissue implants. Functionalized cellulose membranes have been used for heavy-metal ion adsorption from aqueous solutions. Wei et al. introduced polyhydroxy functional groups on the regenerated cellulose membrane using poly(glycidyl methacrylate) and N-methylglucamine to remove boron from aqueous solutions. The authors reported maximum boron adsorption of 0.75 mmol/g under neutral pH [161]. Wittmar and Ulbricht prepared catalytically active porous TiO_2_-cellulose self-cleaning nanocomposite films with potential applications in water purification [162]. Bedane et al. recommended surface coating or chemical modifications of cellulose films for improving films’ moisture barrier properties, especially in highly humid environmental conditions [156] because surface hydrophilicity of films increases due to glycerol plasticization. Rumi et al. showed that hydrophobicity can be imparted into glycerol plasticized and hot-pressed cellulose films via plasma induced surface grafting of oleic acid [159].

### 3.3. Surface Modification of Cellulose Nanomaterials 

Nanocellulose/cellulose nanomaterials (CNMs), nanosized cellulose materials derived often from wood pulp, are also an important class of cellulose with remarkable mechanical, and film forming properties. These materials possess at least one dimension in nanometer range [163]. Owing to the highly ordered organization of cellulose polymer chains into fibrils during biosynthesis, nanoparticles of cellulose can be subsequently extracted [164]. Different from conventional cellulose materials, CNMs possess unique characteristics, such as nano-scale size, special morphology and geometrical shapes, high aspect ratio, crystallinity, high specific surface area, impressive stiffness and strength, dimensional stability, liquid crystalline behavior, alignment and orientation, low coefficient of thermal expansion, and surface chemistry tunability [165,166]. These features combined with abundance and renewability of cellulose make CNMs an important class of futuristic materials with several potential commercial applications including nanocomposite materials, biomedical products, cements, food packaging, antimicrobial materials, energy storage (e.g., supercapacitors), and electronics devices [8,167,168,169,170,171,172]. 

**Figure 8:**
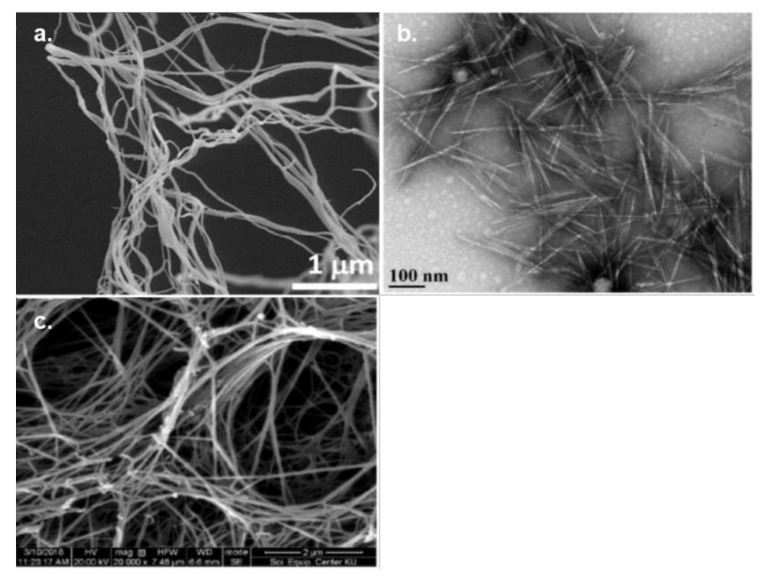
Different types of nanomaterials: (**a**) cellulose nanofibrils (CNFs) obtained from mechanical disintegration of pinewood cellulose, (**b**) cellulose nanocrystals (CNCs) obtained from sulfuric acid hydrolysis of filter paper, and (**c**) bacterial cellulose obtained from culture of *Komagataeibacter xylinus*. Reprinted, (**a**) from ref. [182] © 2021 with permission from Elsevier, (**b**) from ref. [181] as distributed by Creative Common CC BY license, (**c**) from ref. [183] © 2021 with permission from Elsevier.

There are three major categories of CNMs: 1) cellulose nanofibrils (CNFs) or nanofibrillated cellulose (NFC), 2) cellulose nanocrystals (CNCs) or cellulose nanowhiskers (CNWs), and 3) bacterial nanocellulose (BC) (Figure 8) [173,174]. CNFs are entangled individual or bundled fibrils released after mechanical breakdown of already purified cellulose substrate. CNFs possess both crystalline and amorphous domains and are 4–20 nm wide and 500–2000 nm long [8]. CNCs are highly crystalline, elongated, and rod-like (or needle-like) nanoparticles extracted by preferential removal of amorphous domains typically by acid hydrolysis treatment. However, they may also contain residual amorphous domains [175]. CNCs extracted from plant-based cellulose sources are 3–5 nm wide and 50–500 nm long [8]. While CNCs and CNFs are obtained by chemical and mechanical breakdown of the native cellulose structure, bacterial cellulose (BC) is produced by some species of bacteria such as *Komagataeibacter xylinus* in the form of microfibrils of rectangular cross-sections (6–10 by 30–50 nm). Further aggregation of these microfibrils leads to entangled ultrafine ribbons (nanofibers) of 20–100 nm in width and micrometers in length, ultimately generating a three-dimensional web-shaped network structure [8,176]. BC has unique valuable properties, including highly porous network structure, high specific surface area, and high water holding capacity [177].

In addition to the presence of reactive hydroxyl groups, nanocellulose possesses a large surface area (i.e., several hundred m^2^/g) and a high ratio of surface area to volume [176]. These attributes impart high reactivity and thus can be modified and functionalized with relative ease. The surface of nanocellulose can be modified by means of oxidation, etherification, esterification, and grafting [178,179,180]. The modifications not only help enhance the processability (e.g., dispersion in solvents) and compatibility (e.g., when mixing with other polymer matrices) of nanocellulose [15,165,181], but can also impart beneficial functionalities such as antimicrobial properties [170]. 

#### 3.3.1. Cellulose Nanofibrils (CNFs)

CNFs are produced when cellulose fibers are cleaved in the transverse direction as a result of mechanical shearing, leading to the destruction of native hierarchical structure and subsequent release of long and flexible individual or bundle of fibrils [8]. Typically, for the isolation of CNFs, a dilute suspension of cellulose fibers (cellulose slurry) is subjected to high shear forces and various mechanical processes, such as high pressure homogenization (HPH), microfluidization, grinding, cryocrushing, and high-intensity ultrasonication [184]. These mechanical processes are energy intensive. For example, multiple cycles of shearing at high pressure (up to 150 MPa) in the case of HPH and microfludization are required to obtain CNFs of high homogeneity [185,186]. Therefore, usually, various chemically mediated oxidation (e.g., 2,2,6,6-tetramethyl-1-piperidinyloxy (TEMPO) and formic acid hydrolysis) and enzymatic pretreatments (e.g., endoglucanases) are applied prior to mechanical defibrillation for producing CNFs [179,187]. These pretreatments help in loosening the fibrillar structure and facilitate swelling of cellulose, ultimately easing mechanical defibrillation and reducing the energy consumption during CNFs preparation [179]. Specific properties of CNFs such as dimensions, aspect ratio, and surface charge are largely governed by the preparation methods employed with a little effect of cellulose source [174]. “Greener” and more efficient pathways for producing CNFs have also been reported. In a recent study, Ramakrishnan and coworkers reported the preparation of CNFs from cotton cellulose by oil-bath heating cellulose in glycerol at ~180–200 °C [168]. The authors reported an impressive CNFs yield of 71%. 

Surface-modified CNFs are currently receiving considerable attention. Mou et al. performed selective oxidation of CNFs at C2–C3 position using periodate oxidation method (NaIO_4_) to obtain 2,3-dialdehyde nanocellulose [188]. The authors reported that the produced dialdehyde nanocellulose showed good inhibition activity against *Staphylococcus aureus* and more obnoxious methicillin-resistant *Staphylococcus aureus* (MRSA) while showing biocompatibility with mammalian cells. Another interesting study showed the importance of the surface modification of nanocellulose comprised of simple oxidation. Weishaupt et al. employed TEMPO-mediated oxidation for preparing surface modified CNFs with negatively charged carboxylic groups [189], and adsorbed positively charged nisin peptide, known for its bioactivity, onto the negatively charged surface modified CNF. The CNFs-nisin composite exhibited antimicrobial activity against *Staphylococcus aureus*. The authors reported that complete inhibition was achieved at two times lower dose of CNFs-nisin composite compared to that of neat nisin solution. In a recent study, the antimicrobial potential of chemically modified CNFs was investigated by Hassanpour and coworkers [190]. The investigators first modified the surface of the CNFs by covalent attachment of (3-trimethoxysilylpropyl)phenanthridinium iodide (TMSPhI), a quaternary ammonium salt, at different weight percentages (10–100 wt% TMSPhI with respect to CNFs). The modified CNFs containing 100 wt% of TMSPhI (CNFs-TMSPHI) showed strong antibacterial activities against *S. aureus* and *E. coli*. The antibacterial effect of the modified nanocellulose was more pronounced against *E. coli* than against *S. aureus*. The authors also reported that the modified nanocellulose (CNFs-TMSPHI) was not cytotoxic at low concentrations towards normal cells, indicating potential suitability in biomedical applications such as wound healing bandages. 

Surface modifications of CNFs have been performed for other applications, such as adsorption. He et al. prepared modified CNFs by covalently grafting quaternary ammonium functional groups using 2,3-epoxypropyltrimethylammonium chloride [136]. The functionalized CNFs were later used in the preparation of aerogels for heavy-metal ion removal. The authors reported that the aerogels prepared from functionalized CNFs were highly effective in removal of Cr(VI) from water. Only one gram of aerogel adsorbent prepared from modified CNFs was sufficient in removing 99% of Cr(VI) from 1 L solution at a concentration of 1 mg/L in less than 1 h, while the adsorption efficiency of the aerogel prepared from unmodified CNFs was only 84% even after 3 h. 

#### 3.3.2. Cellulose Nanocrystals (CNCs)

Cellulose nanocrystals are typically prepared by selective removal of amorphous domains of cellulose using acid hydrolysis of purified cellulose materials (e.g., scoured and bleached wood pulp and cotton fibers). Sulfuric acid is widely used for preparing CNCs [191]. Although slight variations in hydrolysis conditions are common [192,193,194], CNCs are typically prepared by hydrolyzing the cellulose substrate with ~64 wt% sulfuric acid for 1 h at 45–50 °C [175], although the time of the hydrolysis reaction can vary based on the temperature [164]. When native cellulose fibers are subjected to sulfuric acid hydrolysis, cellulose chains in amorphous regions of microfibrils are selectively hydrolyzed as the chains in the amorphous regions are readily accessed by the acid. Consequently, this process releases individual CNCs [195]. Although sulfuric acid hydrolysis remains as the method of choice for the preparation of CNCs, several other inorganic as well as organic acids such as hydrochloric acid, nitric acid, hydrobromic acid, phosphoric acid, oxalic acid, maleic acid, citric acid, and mixture of acids have been successfully employed to prepare CNCs [196]. In addition to acid hydrolysis, studies have shown that other chemical methods such as TEMPO-mediated oxidation [197], periodate oxidation [198], and oxidation with other common oxidizing agents (e.g., ammonium persulfate and potassium permanganate) [199,200] can be exploited to prepare CNCs from cellulose substrates. Moreover, CNCs can also be prepared using certain ionic liquids such as 1-butyl-3-methylimidazolium hydrogen sulfate and 1-ethyl-3-methylimidazolium acetate [201,202]. Apart from various chemical methods, researchers have successfully prepared CNCs by environmentally friendly enzymatic hydrolysis of cellulose using endo-*β*-1,4-glucanase (endoglucanase, EG) [203,204]. However, chemical methods are more efficient than biological ones in terms of time and yield [196]. Unlike CNFs, whose properties mostly depend on the preparation method with little effect of the cellulose source, properties of CNCs such as morphology and other functional properties (e.g., thermal and colloidal stability) are affected by both the cellulose source and the method of preparation [205,206]. For instance, CNCs derived from tunicates have a length of several micrometers with aspect ratio of approximately 70, while CNCs obtained from lignocellulosic biomass possess lower aspect ratios of 10–30 and are spherical or shorter rod shaped [207]. In general, CNCs prepared by hydrolysis with sulfuric acid under shorter reaction time are larger in size and exhibit enhanced thermal stability but have reduced colloidal stability and vice-versa. 

Because of their impressive mechanical properties, CNCs have a great potential as reinforcement fillers for composite materials and have been widely studied accordingly. However, the hydrophilic nature and the high surface charge of CNCs pose challenges in their dispersion in nonpolar solvents and polymer matrices, including water-based polymers [208]. Modifications to impart hydrophobic characteristics help overcome these challenges. Kaboorani and Riedl carried out surface modification of CNCs by physically adsorbing hexadecyltrimethylammonium bromide (HDTMA), a cationic surfactant, and imparted hydrophobicity to the modified CNCs [209]. CNCs modified with higher concentration of HDTMA under longer reaction time showed a good dispersion in tetrahydrofuran. In another study, modified CNCs were prepared by grafting octadecyl isocynate. The grafted CNCs were later used as fillers in nanocomposites of poly (butylene adipate-co-terephthalate) (PBAT) matrices [210]. The authors reported that while the unmodified CNCs were agglomerated and poorly dispersed in PBAT, the CNCs-containing nanocomposite were uniformly dispersed in PBAT (confirmed by ~ 30% increase in elongation at break as compared to the neat PBAT). The authors also observed increased biodegradation of nanocomposites as compared to the neat BPAT. Ogunsona et al. prepared modified CNCs by surface grafting of acrylonitrile butadiene rubber and investigated their applications in nanocomposites with polylactic acid (PLA) [211]. The authors reported that acrylonitrile butadiene rubber grafted CNCs exhibited improved dispersion in PLA, which translated into 25% higher tensile strength of the nanocomposite containing modified CNCs as compared to neat PLA, whereas nanocomposite of PLA containing unmodified CNCs had only 7% increase in tensile strength. 

Apart from well-established applications such as reinforcement fillers as discussed earlier, CNCs are emerging as efficient stabilizers of Pickering emulsion systems [212]. Tang et al. modified the surface of CNCs by grafting Poly [2-(dimethylamino)ethyl methacrylate] using free radical polymerization and investigated oil-water emulsion stabilization efficiency of the modified CNCs [66]. The authors observed improved stabilization by modified CNCs as compared to unmodified CNCs. The modified CNCs in the emulsion system were pH and thermal responsive, making them suitable for cosmetic and pharmaceutical applications.

#### 3.3.3. Bacterial Nanocellulose (BC)

The methods of preparing bacterial nanocellulose are fundamentally different from the preparation of CNCs and CNFs. CNFs and CNCs are prepared by using a “top-down” approach, where cellulose fibers of higher dimensions are broken down to nanosized particles using mechanical, chemical, or enzymatic methods. Conversely, BC is prepared by a “bottom-up approach”, where glucose molecules are metabolized by certain bacterial strains to construct a 3D network structure of cellulose microfibrils as their extracellular matrix during fermentation [174,213]. Bacterial strains capable of producing nanocellulose belong to several genera, including *Komagateibacter*, *Alcaligens*, *Agrobacterium*, *Rhizobium*, *Xanthococcus*, *Pseudomonas*, *Azotobacter*, and *Aerobacter*. However, owing to high yield, *Komagataeibacter xylinus* is the most widely used species for BC production [213,214]. Typically, BC in the form of pellicle is obtained by incubating a suitable bacterial strain in glucose-rich culture medium for several days. Numerous factors such as bacterial strains, culture medium, culture conditions (e.g., pH of the medium, static or agitated cultivation), and the duration of cultivation affect the yield and specific properties of BC [215,216]. The fibrous network of BC can also be used as a substrate for the production of bacterial cellulose nanocrystals by acid hydrolysis [205,217] or other measures [218]. 

Despite the high production cost and lower production rate, adoption of BC may be feasible in specialized applications such as in biomedical and pharmacological fields [219,220], because of desirable properties of BC such as high mechanical strength, highly porous network structure, and high purity. Badshah et al. investigated controlled drug release properties of acetylated surface-modified BC matrices [221]. The authors showed that the drug-loaded acetylated freeze-dried BC matrices showed improved controlled drug release properties under in vitro drug settings compared to those of unmodified BC matrices. In another production method of functionalized BC, Gao et al. demonstrated in situ functionalization of BC during biosynthesis [222]. The authors were able to obtain BC with unnatural characteristic fluorescence by culturing *Komagataeibacter sucrofermentans* in a medium containing chemically modified glucose (6-carboxyfluorescein-modified glucose). The bacteria metabolized modified glucose units and ultimately imparted fluorescence to the BC.

## 4. Conclusions and Future Perspectives 

Cellulose-based materials can be prepared from a variety of sources: native cellulose (including bacterial cellulose), cellulose composites with other (bio)polymers, cellulose derivatives (e.g., cellulose acetate, methyl cellulose), and cellulose-organic/inorganic hybrids, and their potential applications are countless. Nonetheless, in a highly competitive global market, it is *high-value* applications that most significantly affect market growth. 

In this regard, high-value biotechnological applications that take advantage of cellulose biocompatibility and biodegradability are of interest. Namely, cellulose is used for manufacturing of delivery vehicles for drugs, proteins, and antibodies, scaffolds, sutures, wound healing dressings, etc. For instance, cellulose-based drug excipients, which are able to modify the solubility of drugs and induce different mechanisms of drug release, are used as sustained drug delivery systems [223]. Nanofibrous cellulose derivatives are used as skin substitutes for wound treatment applications [224,225,226]. Antimicrobial nanoparticles/cellulose composites (in form of membranes) have been prepared for slow-release of actives from antibacterial wound dressing [227,228]. Recently, proteins were immobilized into cellulosic aerogel through a water-based self-assembly and used in pH-controlled release [227]. Cellulose-based hydrogels are immensely important for tissue engineering [228], because nanostructured cellulosic materials allow for cellular adhesion [229]. Cellulose templates have been used as artificial blood vessels for modeling tumor invasion in vitro (e.g., cellulose-based microtubes using a chitosan sacrificial template [230]). 

There are countless examples, and the value of surface modification of cellulose through either covalent or ionic functionalization cannot be overstated: such tailoring of surface properties of the manufactured materials allows integration of unique material characteristics without affecting the beneficial properties of the bulk cellulose (renewability, biodegradability, biocompatibility, and strength). This approach stimulates adoption of sustainable technologies and allows for wise utilization of resources, resulting in adaptation of sustainable practices.
